# Increases in Strain, Strain Rate, Displacement and Velocity in the Thoracic Aorta After Bench Pressing

**DOI:** 10.3390/medicina61111950

**Published:** 2025-10-30

**Authors:** María Belén Martínez-Lechuga, Javier Hidalgo-Martín, Manuel Ruiz-Bailén

**Affiliations:** 1 Jaén University Hospital, 23007 Jaén, Spain; 2 Cardiac Care Unit, Intensive Care Unit, Jaén University Hospital, 23007 Jaén, Spain; 3 Department of Health Sciences, Faculty of Health Sciences, Campus Las Lagunillas, University of Jaén, 23071 Jaén, Spain

**Keywords:** speckle tracking, 3D echocardiography, athlete, bench press, aorta artery

## Abstract

*Background and Objectives:* This study aimed to investigate changes in the descending thoracic aorta (DTA) values in athletes while performing a bench press exercise, and to evaluate their relationship with vitamin levels and nutritional values. *Materials and Methods*: The study used speckle tracking to assess changes in DTA parameters in athletes before and after bench press exercise, compared to non-athlete controls. Measurements included rotational and radial velocities, circumferential strain and strain rate, and displacement. *Results:* The study included 60 non-athlete controls and 178 athletes performing bench press exercises. In a 10-year follow-up of 30 weightlifters, aortic speckle tracking values were age-matched with controls. No significant baseline differences were observed between groups. However, following exercise, all measurements demonstrated increases: DTA rotational velocity (55.44 ± 16.15 vs. 88.98 ± 10.31°/s), radial velocity (1.02 ± 0.36 vs. 1.56 ± 0.42 cm/s), circumferential strain (−8.52 ± 0.31 vs. −12.55 ± 1.13), strain rate (−1.55 ± 0.72 vs. −2.28 ± 0.56 s^−1^), rotational displacement (6.22 ± 0.36 vs. 14.91 ± 0.85°), and radial displacement (0.89 ± 0.31 vs. 1.19 ± 0.65 mm), with all *p*-values < 0.05. Rotational displacement correlated with maximal repetition (88.56 ± 12.59 Kg) and vitamin levels. *Conclusions:* This study hypothesizes that bench press exercise is linked to increased DTA velocities, strain, strain rate, and displacement.

## 1. Introduction

Exercise supports cardiovascular health and can improve vascular conditions [1]. Physical activity may affect aortic elasticity and structure, and the sport type may influence aorta size [2,3,4,5]. Additionally, various sports have been proposed to impact aortic dimensions, which may be associated with outcomes such as aneurysms or atherosclerosis [6,7,8,9,10].

In the echocardiographic evaluation of athletes, measurement of aortic diameter remains the principal parameter assessed, with magnetic resonance imaging providing further detailed analysis [2]. Cardiovascular pathologists frequently direct their attention primarily toward embolic risk and atherosclerosis, which may result in comparatively less focu/s on the nuances of aortic pathology. Patients exhibiting aortic dilation are subjected to comprehensive surveillance, with interventions implemented during episodes of acute aortic syndrome. Emerging research emphasizes the necessity of expanding our knowledge of aortic physiology and understanding its broader impact on cardiovascular health.

Speckle tracking is an echocardiographic imaging technique commonly used to assess systolic function and prognosis in aortic valve disease [10]. Speckle tracking echocardiography allows for the measurement of strain, strain rate, displacement, and velocity of ventricular fibers in comparison to baseline values. This approach has contributed to a better understanding of both the physiology of sports practitioners and various pathologies. This technique, while advantageous, is rarely used to assess the aorta in athletes. Although ventricular fiber strain and velocity rise during exercise, normal reference values—especially for athletes—are still unknown [11]. At least 40% of Americans and Europeans participate in sports (https://www.ine.es, accessed on 28 October 2025). Most studies focus on endurance activi/ties like running and swimming, with less attention given to strength sports, especially weightlifting [1,2,3,4,5,6,7,8,9,10,11,12,13]. Although a significant population participates in these athletic activities, there is a scarcity of research assessing their cardiovascular responses [14]. Physical activity may induce alterations in the aorta, with such changes potentially differing by sport type [14]. To date, no research has directly assessed aortic function or behavior in this or any other sport.

Nutritional and vitamin status influences myocardial function. The association between the nutritional profiles of strength athletes and their cardiac remodeling remains unresolved and may potentially affect the functional properties of the thoracic aorta [15,16].

A recent evaluation of speckle tracking velocity vector analysis (VVI) in the thoracic aorta of healthy individuals has led to proposed reference values [17]. These values can differ in athletes and may vary with physical activity. Speckle tracking is more sensitive than traditional echocardiography and may relate to nutritional parameters.

Our hypothesis is that bench press exercise induces acute increases in the parameters assessed by speckle tracking, particularly velocities, displacement, and strain of the descending thoracic aorta (DTA). Furthermore, we hypothesize that these echocardiographic measurements possess sufficient sensitivity to correlate with nutritional values.

This study aimed to establish baseline values for strain, strain rate, velocities, and displacement of the DTA, and to assess their changes after bench press exercise. Additionally, we examined correlations between echocardiographic and nutritional variables and evaluated cardiac biomarker release after training.

## 2. Materials and Methods

**Design of the study:** This is an observational study evaluating echocardiographic parameters of the thoracic aorta before and after performing a bench press exercise. The study was conducted from 2014 through January 2024; however, participants were not included between 2020 and 2022 due to the COVID-19 pandemic. Baseline transthoracic echocardiography (TTE) was conducted before and after bench press exercise using Siemens SC 2000 and Sequoia 512c systems with Syngo software 2013 version (Mountain, CA, USA) Although the software is designed for ventricles, it was used to assess the aorta, which may introduce bias.

Thirty athletes enrolled in 2014 were followed for 10 years and re-evaluated with another bench press in 2024. All participants gave informed consent, data protection laws were observed, and the study adhered to the Declaration of Helsinki as well as applicable legal and regulatory requirements.

**Figure 1:**
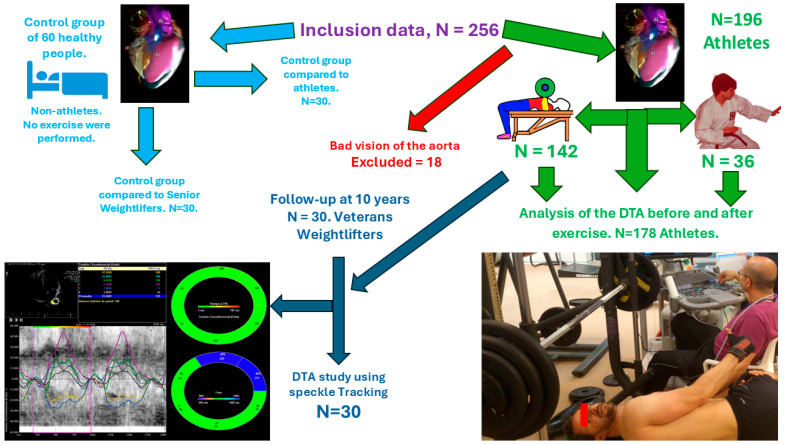
The study included 256 healthy participants, separated into two groups. The first group was comprised of 196 athletes; however, 18 were excluded due to difficulties with thoracic aorta visualization, resulting in a final count of 178 athletes. The second group consisted of 60 healthy men with no history of strength or endurance exercise. Of these, 30 were compared to the 178 athletes who performed the bench press, and another 30 from the control group were compared with athletes assessed at a 10-year follow-up.

**Subject.** Participation in the study was voluntary, beginning with 256 individuals. Participants were divided into two groups: a control group of 60 non-athletes who did not exercise, and an athlete group initially consisting of 196 weightlifters who met the inclusion criteria. However, 18 athletes were excluded due to insufficient technical quality in the echocardiography results following the bench press exercise. Finally, 178 weightlifters remained in the athlete group, of whom 36 also participated in karate (see Figure 1).

**Inclusion criteria.** The male athletes included in the study were between 20 and 45 years of age. We defined athletes as individuals who scored a high level of physical activity (quantitative value > 3000 MET/min per week) on the International Physical Activity Questionnaire (IPAQ; Spanish version April 2002).

**Exclusion criteria.** 1. Having any known cardiovascular disease or having it detected during the study. 2. Poor ultrasound quality during measurement. 3. Not wishing to be included in the study, and therefore not signing the informed consent form. 4. Having been previously diagnosed with arrhythmias. 5. Having been diagnosed with hypertension.

**Control group.** The control group consisted of 60 untrained males who did not engage in bench press or other strength exercises. This group received comprehensive echocardiographic assessments, including advanced imaging modalities, without performing bench press exercises; the main aim was to establish reference values for echocardiographic parameters at the institution. Echocardiographic procedures followed American Society of Echocardiography guidelines, with parameters evaluated using speckle tracking velocity vector imaging (VVI) on the DTA. Measurements included right ventricular strain, strain rate, and ejection fraction. Among the 60 participants, 30 individuals served as an age-matched control group in comparison with 178 weightlifters involved in bench press activities, while the other 30 were matched by age and compared with weightlifters observed over a 10-year period (Figure 1).

### 2.1. Clinical Cohort

All athletes meeting the inclusion criteria underwent transthoracic echocardiography (TTE), with results recorded in high-quality digital format. A blind, off-line analysis was carried out using the Syngo software, Siemens^®^ U.S. 2013 (Mountain, CA, USA).

Blood samples from 68 of the 178 included athletes who consented were analyzed for inflammation, myocardial, and renal injury markers, including troponin I, brain natriuretic peptide, interleukin 6, C-reactive protein, and creatinine. Nutritional status was also assessed by measuring vitamin levels (B1, B3, B6, C, and D), folic acid, magnesium, zinc, and polyunsaturated fatty acids. Samples were taken 6–8 h post-exercise.

### 2.2. Image Acquisition and Processing

Standard transthoracic echocardiograms were performed using a commercially available system, SC 2000 de Siemens^®^ (Mountain, CA, USA) and Sequoia 512c (Mountain View, CA, USA). Transthoracic examinations were performed in the supine position. Left ventricular functional data were acquired in the apical four-chamber orientation. The projections used in the examination of the aorta were the long-axis parasternal and the apical four-chamber with zoom. The region of interest was manually traced along the edge of the intima of the thoracic aorta. The frame rate was as high as possible (70–120 f/s), with multiple focal points. All images were optimized using gain, compression, and dynamic range. We used probes 4V1C and 4Z1c. A blinded, off-line analysis was performed without knowledge of the individual’s name or physical condition, using Syngo software (Siemens^®^, Mountain View, CA, USA, 2013).

We evaluated the usual echocardiographic parameters of the American Society of Echocardiography. We calculated fractional shortening area and speckle tracking-derived parameters such as strain (S), strain rate (SR), displacement, and longitudinal and radial velocities in the thoracic aorta.

### 2.3. Bench Press Exercise

Measurements were collected before and after exercise. The maximum load for 1 RM was calculated one week prior. Most bench press tests took place in the afternoon. To assess chest strength and reduce injury risk, participants followed the protocol in Table 1. After this exercise (series and rest included), an echocardiography was performed.

### 2.4. Statistical Analysis

This study aimed to explore and generate new hypotheses. Variable normality was verified with the Kolmogorov–Smirnov test, followed by ANOVA and Student’s *t*-test for quantitative analysis, and Levene’s test for homogeneity of variance. Bonferroni and Tukey post hoc tests were used when variances matched to control Type I error. No multivariate analyses were performed, focusing solely on pre- and post-exercise DTA parameter changes via Speckle Tracking VVI. Pearson’s correlation assessed cardiac metrics, and Bland–Altman plots plus logistic regression examined agreement between imaging planes. Interobserver and intraobserver reliability for DTA metrics was evaluated. Results are presented as means ± SD with significance at *p*  <  0.05. Analyses were conducted using IBM SPSS 29 and MedCalc Software Ltd. Ostend, Belgium.

## 3. Results

### 3.1. The Control Group and Athletes at the Beginning of the Study

30 non-athletic healthy participants in the control group (mean age 35.27 ± 8.22 years) and 178 males in the bench press group (mean age 33.47 ± 9.21 years; *p* > 0.05) were compared. Prior to exercise, no significant differences were found between groups regarding DTA Speckle Tracking values or diameter (26.37 ± 3.57 mm; *p* > 0.05, Student’s *t*-test). The average one-repetition maximum (1 RM) load was 88.56 ± 12.59 kg (Figure 1).

**Figure 2:**
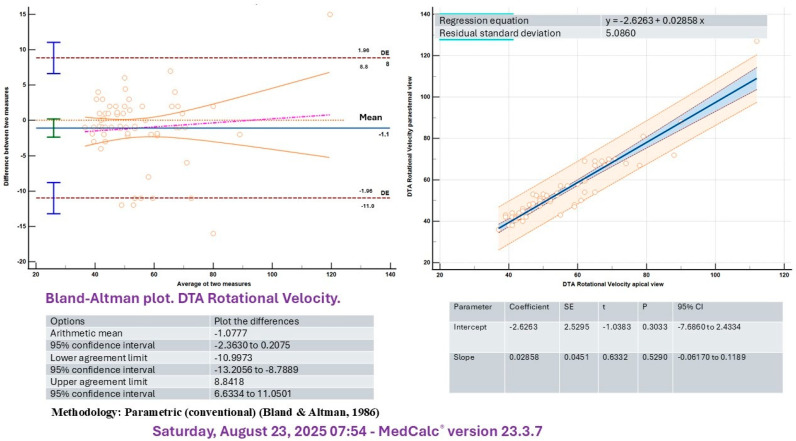
Bland–Altman plot for DTA rotational velocity.

**Figure 3:**
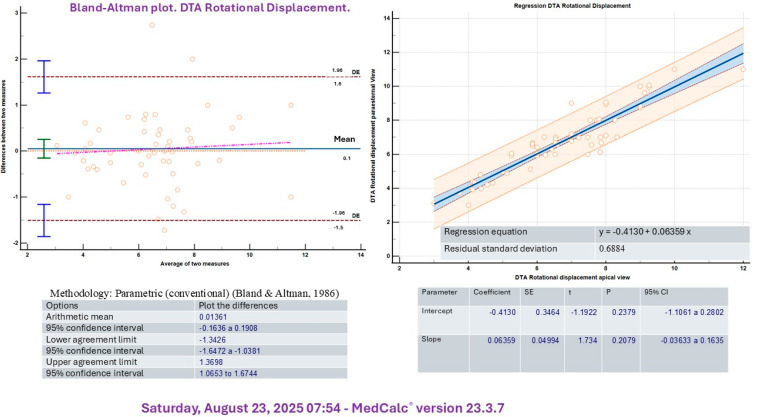
Bland–Altman plot for DTA rotational displacement.

A significant correlation was identified between measurements obtained by two independent echocardiographers for both rotational velocity (R^2^ = 0.81, *p* < 0.005) and rotational displacement (R^2^ = 0.86, *p* < 0.05) of the DTA. In addition, intraobserver analysis demonstrated similar correlations for rotational velocity (R^2^ = 0.76, *p* < 0.05) and rotational displacement (R^2^ = 0.78, *p* < 0.05) of the DTA. Although aortic parameters were primarily assessed in the parasternal short-axis view, the control group also underwent evaluation using the apical four-chamber projection. The Bland–Altman analysis demonstrated satisfactory agreement between the apical four-chamber and parasternal long-axis projections for both rotational velocity and displacement of the DTA (Figure 2 and Figure 3).

### 3.2. Differences Between Before and After Bench Press Exercise

Although physiological changes reached statistical significance, they were not considered clinically relevant (Table 2). Blood counts, biochemical parameters, and troponin levels remained within normal ranges.

The ANOVA test conducted to compare data from 30 individuals in the control group and 178 athletes before and after performing the bench press suggests that cardiac function measurements in the control group may be higher than those recorded at baseline in the athlete group (Table 3).

No major differences were observed between the control group and baseline athletes in Speckle Tracking DTA measurements using Student’s test. Fractional shortening DTA area measured 0.45 ± 1.21%, rotational velocity DTA was 54.55 ± 0.18°/s, radial velocity DTA reached 1.12 ± 0.23 cm/s, circumferential strain DTA registered −8.88 ± 0.45%, circumferential strain rate DTA came to −1.45 ± 0.25/s, rotational displacement DTA was 8.22 ± 1.75°, and radial displacement DTA was 0.87 ± 0.28 mm. All *p*-values < 0.05.

**Figure 4:**
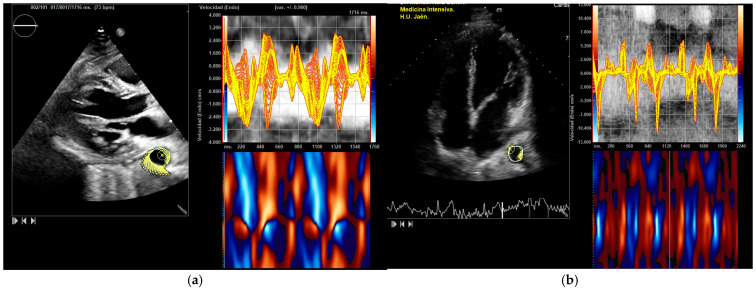
DTA vectorial velocity analysis. Parasternal and apical four-chamber views. The aortic border contour is drawn and explored using speckle tracking. The image displays velocities and the anatomical curved mode: (**a**) parasternal view; (**b**) apical view.

**Figure 5:**
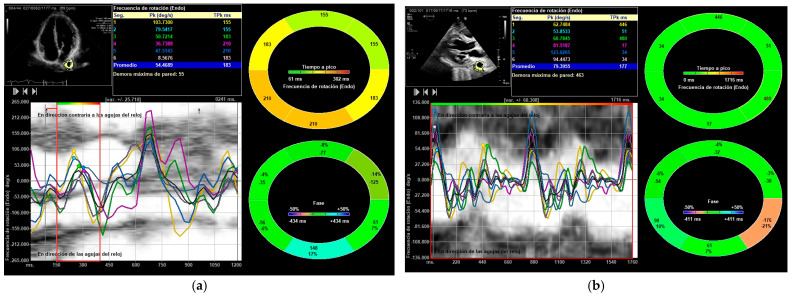
“Frecuencia de rotación (Endo)” = Rotational velocity. The thoracic aorta is outlined and divided into six zones, with the systolic peak values displayed. The average systolic peak value is taken. These measurements are reported in degrees per second. In this athlete, values rose from 54°/s to 79°/s after exercise: (**a**) DTA rotational velocity before exercise; (**b**) DTA rotational velocity after exercise.

**Figure 6:**
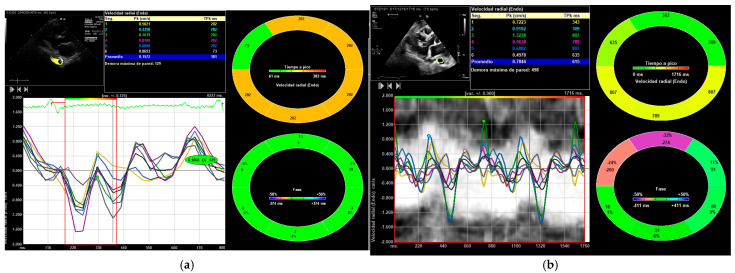
Velocidad radial (Endo) = Radial velocity. The DTA radial velocity (cm/s): (**a**) DTA radial velocity before exercise; (**b**) DTA radial velocity after exercise.

All parameters showed an increase following exercise (Table 2, Table 3 and Table 4): DTA rotational velocity (55.44 ± 16.15 vs. 88.98 ± 10.31°/s), radial velocity (1.02 ± 0.36 vs. 1.56 ± 0.42 cm/s), circumferential strain (−8.52 ± 0.31 vs. −12.55 ± 1.13), strain rate (−1.55 ± 0.72 vs. −2.28 ± 0.56 s^−1^), rotational displacement (6.22 ± 0.36 vs. 14.91 ± 0.85°), and radial displacement (0.89 ± 0.31 vs. 1.19 ± 0.65 mm), with all *p*-values < 0.05 (Figure 4, Figure 5 and Figure 6). RM [88.56 ± 12.59 Kg] correlated with DTA rotational displacement (Pearson’s r 0.41, *p* < 0.05).

### 3.3. Karate Subgroup

Among the 178 weightlifters, 36 participants trained in Shotokan Karate and had experience in strength and Kumite combat. Differences between before and after bench press exercise were evaluated within this subgroup. The karate practitioners demonstrated higher longitudinal strain of the basal left and global right ventricles (−23.74 ± 12.32 and −30.28 ± 13.22, *p* < 0.05 for both) and exhibited a greater post-exercise increase in these strains (−32.84 ± 22.21 and −35.45 ± 18.25, *p* = 0.032 and *p* = 0.022, respectively). In the DTA, significant changes after the bench press were observed in rotational velocity (105.14 ± 25.47°/s, *p* = 0.04, Student’s *t*-test) and rotational displacement (15.41 ± 6.54°, *p* < 0.005), with both measures increasing more among karate practitioners compared to non-karateka.

### 3.4. Follow-Up at 10 Years

Thirty athletes who participated in the study in 2014 underwent a follow-up echocardiography in January 2024. Their mean age was 42.82 ± 11.23 years, compared to the mean age of 41.21 ± 13.18 years for the 30 non-athlete individuals in the control group (*p* = 0.348). After ten years of strength training, speckle tracking values in DTA did not show significant differences; all *p*-values were greater than 0.05. DTA rotational displacement increased from 6.18 ± 1.15° to 8.89 ± 1.88° (*p* = 0.008). Statistical analyses were conducted using Student’s *t*-test.

### 3.5. Nutrition and DTA Speckle Tracking

The DTA rotational velocity was correlated with the level of proteins (Pearson’s r 0.58, *p* value = 0.032)), with the level de zinc (Pearson’s r 0.46, *p* value = 0.0001) and with vitamins B1 (Pearson’s r 0.56, *p* value 0.0001), B3 (Pearson’s r 0.48, *p* value = 0.028 and B6 (Pearson’s r 0.36, *p* value = 0.001). All athletes took nutritional supplements. The rest of the biochemical and analytical study was normal. The rotational displacement correlates con vitamins B1 (Pearson’s r 0.45, *p* value 0.0001), B3 (Pearson’s r 0.87, *p* value = 0.001 and B6 (Pearson’s r 0.69, *p* value = 0.001), and vitamin C (Pearson’s r 0.56, *p* value 0.001).

## 4. Discussion

Recent research indicates that the function and injury of the aorta play a significant role in modulating cardiovascular responses. Additionally, studies have demonstrated that aldosterone inhibitors are effective in decreasing aortic stiffness [18]. Additionally, levosimendan may relax the muscle of the aorta [19]. Furthermore, a clear association exists between cardiovascular function and aortic elasticity, and the structural integrity of the medial layer not only affects aortic stiffness but also impacts clinical prognosis [20,21]. Zapolski observed reverse remodeling and altered stiffness in transplant recipients. Athletes often show benefits, but may also experience greater aortic dilatation than others, though its clinical significance is uncertain [22]. Current diagnostic approaches for the aorta are primarily focused on measuring diameter or identifying atheroma as indicators of disease [23]. Although speckle tracking has contributed to understanding cardiac physiology, research on its application to the aorta remains limited. Study results vary, but all suggest this tool may improve aortic research. Recently, we used Speckle Tracking VVI [11] to assess aortic parameters in healthy individuals and ventricular strain in weightlifters. Whether these parameters differ by sport or disease is still unknown [17,24,25,26]. This study evaluates normal speckle tracking parameters in healthy individuals and weightlifters, comparing DTA behavior changes among non-athletes and athletes before and after exercise. The findings support the hypothesis that athletes uniformly exhibit an increase in aortic parameters following exercise. Additionally, ten years of longitudinal monitoring in athletes has consistently revealed sustained elevations in speckle tracking echocardiographic parameters post-exercise, suggesting the hypothesis of cardiac remodeling associated with aging. These new hypotheses are clinically relevant, suggesting that aortic parameters measured with speckle tracking may vary according to exercise, activity type, or certain pathologies. These results suggest that this technological tool could be applicable in DTA for various sports and in clinical medicine by helping to identify patterns associated with certain diseases. Furthermore, our findings are consistent with studies that report an improvement in aortic elasticity with training. The increases in deformation observed may be attributed to greater adaptive aortic elasticity because of sporting activity [3,4,5].

In this study, we evaluated basal DTA values exclusively in a population of young men and found a high level of concordance between measurements obtained from the long-axis and apical four-chamber views. Both imaging projections appear to be well-suited for this assessment.

A minor exploratory correlation was observed in a small population between speckle tracking parameters and nutrition and vitamin levels. However, no causal relationship can be established since no multivariate analysis was performed. Nevertheless, it raises the hypothesis that nutrition might contribute to aortic remodeling [27,28,29,30,31,32].

### 4.1. Clinical Applicability

This study finds that Speckle Tracking VVI is a feasible method for assessing the aorta. DTA rotational velocity, strain, and displacement were correlated with maximum RM, suggesting an association—though not causality—between these metrics and exercise-induced cardiac and aortic remodeling. While strain is sometimes assumed to be homogeneous throughout the entire aorta, there may be significant regional variability, with each segment having distinct values. In this study, speckle tracking was applied only to the thoracic aorta; measurements could differ in other regions of the vessel. Such measurements may enhance the understanding of aortic pathophysiology and various aortic diseases. Furthermore, they could provide relevant information for endovascular interventions involving the thoracic aorta and conditions such as atherosclerosis, which can alter these parameters and contribute to aortic remodeling or heart failure [7,11,14,18,21,22,23,24,25,26]. Future studies could examine the impact of these parameters on specific cardiovascular diseases or other conditions, such as obstructive sleep apnoea [33,34].

The main implication of this study is the formulation of a hypothesis for subsequent research. Speckle tracking may serve as a valuable tool for assessing the aorta, with the potential to correlate these measurements with sport, aortic pathologies, cardiovascular diseases, and other conditions, such as metabolic disorders.

### 4.2. Limitations

These findings should be considered with caution, as the study involved a limited sample of young men, which constrains the applicability of the results to other age groups or to women. Due to the study design and the lack of multivariable analysis, this research is exploratory in nature; thus, causality cannot be established, and our findings should be viewed as preliminary hypotheses for future investigation. The software employed was developed specifically for ventricular parameters [25,35], and it is important to acknowledge that potential software-related artifacts may have influenced the outcomes and introduced bias, since the program was not specifically designed for aortic analysis. This limitation could account for data loss and difficulties in detecting parameters such as aortic strain, strain rate, or radial measurements—particularly after exercise, when the acoustic window becomes less clear. The application of ventricular software to aortic evaluation may yield results that differ from those obtained with tools designed explicitly for aortic assessment.

One proposed hypothesis is based on a 10-year follow-up study involving a small cohort (*n* = 30), which compared aortic echocardiographic measurements without controlling for potential confounders such as physical fitness or nutrition. The results may suggest that older weightlifters have stable aortic measurements, possibly indicating positive remodeling of the aorta. However, because there were only a few older participants, more research is needed to see if these findings apply to people over 50 or 60 and to better understand any cardiovascular benefits.

We also assessed weightlifters who participate in combat karate (kumite). While these results could be due to a type I statistical error, they propose that the aorta may undergo sport-specific remodeling. However, this hypothesis requires validation through a study specifically designed for this purpose.

This is the first study to evaluate sport-induced changes in the thoracic aorta using speckle tracking. Speckle tracking effectively detects significant aortic changes after strength training, which may result from both ventricular and aortic muscle adaptations. The findings suggest that the aorta, like the heart, undergoes remodeling in response to athletic activity [2,11,14,24,27].

While this study has limitations, its findings have strong clinical relevance. Assessing the aorta provides insight into athletic performance and may reveal changes also seen in cardiovascular disease. Thoracic aorta speckle tracking could therefore be a promising research tool that merits further study.

## 5. Conclusions

This study hypothesizes that bench press increases strain, strain rate, velocity, and displacement of the descending thoracic aorta.

## Figures and Tables

**Table 1:** Steps followed in the bench press exercise.

Step	Instruction
1	Light-load warm-up set, 10 reps
2	Rest 1 min
3	Increase load 5–10%, perform 3–5 reps
4	Rest 2 min
5	Increase load 5–10%, perform 2–3 reps
6	Rest 4 min
7	Increase load 5–10%, perform 1 rep
8	Rest 4 min, increase load 5–10%, repeat
9	If unable to lift, rest 4 min, reduce 2.5–5%, repeat
10	Continue adjusting to find 1 RM
11	After 1 RM: estimate 10 reps
12	Training: 10 sets of 10 reps at 75% 1 RM, 1′30″ break

**Table 2:** Physiological parameters in control and athlete groups. An ANOVA test was used to compare the results obtained among the 30 participants in the control group and 178 athletes before and after performing the bench press. At baseline, the control group demonstrated slightly better cardiac function compared to the athletes. N.S.: not significant.

Variables	Control Group	178 Athletes		*p*-Value
	Do Not Perform Bench Presses (*n* = 30)	Before Bench Press (*n* = 178)	After Bench Press (*n* = 178)	
Systolic blood pressure (mmHg)	117.35 ± 14.56	128.88 ± 09.28	139.78 ± 16.37	<0.05
Diastolic blood pressure (mmHg)	78.11 ± 17.36	88.98 ± 10.31	98.76 ± 25.32	<0.05
Heart rate (B/min)	71.21 ± 13.15	55.14 ± 09.32	110.55 ± 26.41	<0.05
Breathing rate (B/m)	16.38 ± 5.44	15.72 ± 6.87	26.88 ± 6.38	<0.05
SpO_2_ (%)	97.05 ± 0.65	95.42 ± 1.22	98.98 ± 1.24	N.S.

**Table 3:** Biventricular function. Anova test was used to compare the results found among the 30 participants in the control group and the 178 athletes before and after performing the bench press.

Variables	Control Group	178 Athletes		*p*-Value
	Do Not Perform Bench Presses *n* = 30	Before Bench Press N = 178	After Bench Press N = 178	
3D LVEF (%)	65.33 ± 1.19	53.04 ± 1.31	74.78 ± 1.87	<0.05
2D LVEF (%)	56.21 ± 3.22	46.21 ± 1.18	68.44 ± 1.28	<0.05
Right ventricular global longitudinal strain (%)	−27.77 ± 3.27	−23.88 ± 2.88	−30.88 ± 11.02	<0.05
Left ventricular global longitudinal strain (%)	−21.88 ± 4.11	−18.11 ± 3.47	−25.21 ± 3.27	<0.05
Left ventricular global longitudinal rate (1/s)	−1.59 ± 0.93	−1.44 ± 0.45 *	−2.93 ± 0.56 *	<0.05
Right ventricular area shortening fraction (%)	55.78 ± 3.22	47.56 ± 1.28	65.68 ± 2.52	<0.05

LEVF: left ventricular ejection fraction (volumetric methods in apical 4 chambers). * Statistically significant differences.

**Table 4:** DTA speckle tracking values for both basal and post-intervention groups showed significant differences according to Student’s *t*-test (with all *p*-values below 0.05). The average one-repetition maximum (1 RM) was 88.56 ± 12.59 kg (*n* = 178). Each variable result is presented alongside its sample size (N). Due to software detection limitations, fewer participants were evaluated for radial velocity and circumferential measurements. Furthermore, following the bench press exercise, the software’s ability to detect certain variables diminished.

Variable	Before Bench Press	After Bench Press
Right ventricular fractional shortening Area (%)	N = 178; 44.12 ± 1.21	N = 178; 62.36 ± 0.09
DTA rotational velocity (°/s)	N = 178; 55.44 ± 16.15	N = 178; 88.98 ± 10.31
DTA radial velocity (cm/s)	N = 144; 1.02 ± 0.36	N = 144; 1.56 ± 0.42
DTA circumferential strain (%)	N = 149; −8.52 ± 0.31	N = 149; −12.55 ± 1.13
DTA strain-rate circumferential aorta (1/s)	N = 156; −1.55 ± 0.72	N = 138; −2.28 ± 0.56
DTA rotational displacement (°)	N = 168; 6.22 ± 0.36	N = 168; 14.91 ± **0.85**
DTA aorta radial displacement (mm)	N = 178; 0.89 ± 0.31	N = 164; 1.19 ± 0.65

## Data Availability

Data are unavailable due to privacy or ethical restrictions.

## Abbreviations

The following abbreviations are used in this manuscript:

DTA	Descending thoracic aorta
PUFAs	Polyunsaturated fatty acids
S	Strain
SR	Strain rate
RM	Repetition maximal
VVI	Velocity vector analysis
